# Innovative electrochemical electrode modified with Al_2_O_3_ nanoparticle decorated MWCNTs for ultra-trace determination of tamsulosin and solifenacin in human plasma and urine samples and their pharmaceutical dosage form†

**DOI:** 10.1039/d2ra01962k

**Published:** 2022-06-13

**Authors:** Khalid A. M. Attia, Ahmed M. Abdel-Raoof, Ahmed Serag, Sherif M. Eid, Ahmed E. Abbas

**Affiliations:** Pharmaceutical Analytical Chemistry Department, Faculty of Pharmacy, Al-Azhar University Nasr City Cairo Egypt Ahmedmeetyazeed79@yahoo.com Ahmedmeetyazeed79@Azhar.edu.eg; Analytical Chemistry Department, Faculty of Pharmacy, October 6 University 6 October City Giza Egypt

## Abstract

A simple, cheap, sensitive, and time-saving square wave voltammetric (SWV) procedure using a carbon paste electrode modified with aluminum oxide nanoparticle decorated multi-walled carbon nanoparticles (Al_2_O_3_-NPs/MWCNTs/CPE) is presented for the ultra-sensitive determination of tamsulosin (TAM) and solifenacin (SOL), one of the most prescribed pharmaceutical combinations in urology. Characterization of the developed electrode was performed using scanning electron microscopy (SEM), X-ray diffraction (XRD) patterns, energy dispersive X-ray analysis (EDX), transmission electron microscopy (TEM) and FT-IR spectrophotometry. The voltammetric behavior of TAM/SOL was evaluated using Al_2_O_3_-NPs in different content and electrode compositions. The use of Al_2_O_3_ functionalized MWCNTs as a CPE modifier increased the process of electron transfer as well as improved the electrode active surface area therefore, ultra-sensitive results were acquired with a linear range of 10–100 and 12–125 ng ml^−1^ for TAM and SOL respectively, and a limit of the detection value of 2.69 and 3.25 ng ml^−1^ for TAM and SOL, respectively. Interestingly, the proposed method succeeded in quantifying TAM and SOL with acceptable percentage recoveries in dosage forms having diverged concentration ranges and in the biological fluids with very low peak plasma concentration (*C*_max_). Furthermore, the proposed method was validated, according to the ICH criteria, and shown to be accurate and reproducible.

## Abstract 2

A SWV method using a carbon paste electrode modified with aluminum oxide nanoparticle-decorated multi-walled carbon nanoparticles is presented for ultra-sensitive determination of tamsulosin and solifenacin, one of the most prescribed pharmaceutical combinations in urology.

## Graphical abstract

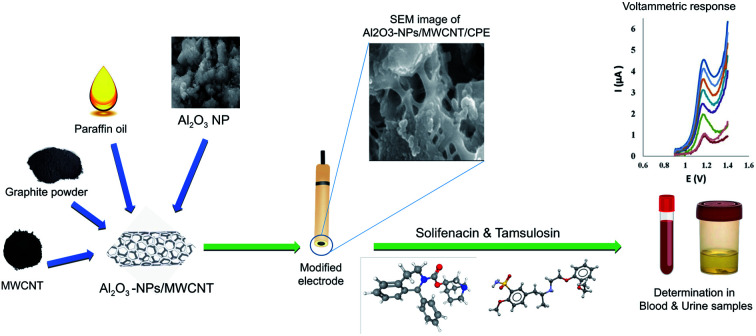

## 1. Introduction

Over the last years, lower urinary tract symptoms (LUTS) were considered the most prevalent urological disorder in men after middle age.^1^ In males, the presence of LUTS was correlated with benign prostatic hyperplasia (BPH).^2^ Intriguingly, US$785 million ($285–301 per patient per year) was estimated to be the cost of fee-for-service (FFS) for BPH/LUTS in the United States, in 2013, excluding medication costs.^3^ Ideally, LUTS can be generally classified into three categories: storage symptoms (*e.g.*, suffering from daytime urinary frequency, and urgency incontinence), voiding symptoms (*e.g.*, suffering from a weak stream (<10 ml s^−1^), and micturition hesitancy), and symptoms of post micturition (*e.g.*, post micturition dribbling, a sense of insufficient bladder emptying). Unfortunately, patient quality of life (QoL) is negatively affected by these symptoms, which are extraordinarily bothersome to patients and disagree with daily life.^4^ Fortunately, several pharmacologic approaches are beneficial for the treatment of LUTS,^5^ including, α-blockers which are extremely assessed as the first-line therapy for male LUTS essentially for management of voiding symptoms such as tamsulosin (TAM), a potent and selective α1A- and α1D-adrenoceptor blocker chemically recognized as [5-(2*R*)-2-[2-[[(2-ethoxyphenoxy)ethyl]amino]propyl]-2-methoxybenzene-1-sulfonamide] which is insoluble in most liquids, including water, glacial acetic acid, ethanol, and ether.^6^ On the other hand, as a first-line treatment for storage symptoms, antimuscarinics are used such as solifenacin (SOL), a selective M3 blocker chemically recognized as [(1*S*)-(3*R*)-1-azabicyclo[2.2.2]oct-3-yl] (1*S*)-1-phenyl-3,4-dihydro-1*H*-isoquinoline-2(1*H*)-carboxylate butanedioate which is soluble in water, methanol, dimethyl sulfoxide, and glacial acetic acid.^7^ Currently, Canadian urological association guideline indicate that adding antimuscarinics to α-blockers increases the ability to management of storage symptoms that bothersomely persist after α-blockers monotherapy.^8^ In addition, several studies prompt the advantages of combination treatment with α-blockers plus antimuscarinics in the improvement of patient quality of life (QoL) and remarkable reduction in bothersome storage symptoms.^9^ Nevertheless, the extensive clinical potential of this combination, there is a difficult obstacle with its use due to the **first-dose phenomenon** (after the first dose is absorbed, a sudden and severe drop in blood pressure can occur, resulting in syncope (fainting)),^10,11^which explain the urgent need for simple, fast, and extremely sensitive analytical methods for quantification of TAM/SOL in biological fluids to help in diagnosis the fainting resulting from the **first-dose phenomenon**.

Intriguingly, the development of a suitable method for simultaneous analysis of TAM/SOL is a challenge because the drugs are present in the ratio of 1 : 15 in their challenging dosage form. Therefore, the relatively wide divergence in the absorptivity and ratio of TAM/SOL became a great challenge for their simultaneous spectrophotometric estimation. In addition, the other greatest obstacle is the simultaneous analysis of TAM/SOL in biological fluids due to their infinitesimal peak plasma concentrations (for TAM equal about 10.1 ng mL^−1^ following breakfast and equal about 17.1 ng mL^−1^ during fasting while for SOL equal 24.0 and 40.6 ng mL^−1^, respectively).^12,13^ The aforementioned challenges explain the drawbacks and difficulties facing the relatively few reported procedures, these include spectrofluorimetric method,^14,15^ spectrophotometric method,^16,17^ HPTLC^18^ and RP-HPLC methods.^19,20^ Furthermore, the in-depth study showed that many other defects were found in each of the aforementioned methods including the requirement for skilled operators, potential drug loss during re-extraction procedure, need for expensive solvents, and influence by the interference of endogenous substances, in addition, these methods require lengthy, and time-consuming processes for preparation and extraction of plasma sample. Furthermore, the need for extremely expensive and sophisticated instrumentation is another concern. Above all, these methods are limited by a high limit of detection and low detection sensitivity, making them ineffective to detect or quantify infinitesimal peak plasma concentrations. So, developing a simple, cost-effective, appropriately selective, and highly sensitive method for TAM/SOL nanomolar determination is still interesting.

In this context, electrochemical detection methods are proved to be a powerful tool to deal with these common challenges as they offer extremely high sensitivity, ease of use, and relatively cost-effective equipment, have a low detection and quantification limit, and are friendly to the environment.^21,22^ The most favorable electrochemical techniques are cyclic voltammetry and square wave voltammetry analysis, with these techniques; we can attain lower detection limits at a lower cost and in less time using facilely prepared and affordable electrodes.^23^ Carbon-based electrodes due to their low background current and wide potential range become a popular choice for electroanalytical methods.^24^ Among several working carbon-based electrodes, the carbon paste electrode (CPE) has been frequently utilized because of its benefits including easy modification, low price, and low ohmic resistance, an extreme potential window, and limited background current.^25^ In addition, carbon nanotubes (CNTs) have been the focus of several studies because of their exceptionally excellent physical, electronic, chemical, structural, and mechanical properties that make them a very desirable material for many applications.^26,27^ Ideally, carbon nanotubes come in two varieties: multiwall (MWCNTs) and single wall (SWCNTs).^28^ MWCNTs have attracted a lot of interest for the fabrication of electrochemical electrodes^29,30^ as normal media do not readily dissolve them. In addition, there have been several proposed binders for MWCNTs incomposite matrices incorporation, including inks,^31^ Teflon,^32^ and mineral oil.^33^ Moreover, the dispersion of MWCNTs in acidic solutions,^34^ such as Nafion,^35^ has been also effectively used. Hence, the use of electrodes modified with MWCNTs allows for the detection of many types of the bioanalytes, such as glucose,^36^ DNA,^37^ and neurotransmitters.^38^

Various nanomaterials have been extensively used as a modifier to improve the performance of the carbon-based electrodes due to their special characteristics.^39^ Among the various nanomaterials, metal oxide nanoparticles are preferred due to their exceptional physical, electronic, and chemical properties,^40–42^ in addition to, better control over nanoparticles deposition,^43^ their chemical stability, and catalytic activity.^44^ Currently, Al_2_O_3_ nanoparticles are preferred among the various metal oxide nanoparticles as aluminum is the cheapest metal having excellent electrical and thermal conductivity.^45,46^ Al_2_O_3_ nanoparticles can be readily synthesized from landfills-accumulated aluminum waste providing an alternative greener waste recycling procedure. On the other hand, Al_2_O_3_ nanoparticles exhibit a great catalytic activity, high stability, and great adsorption capacity.^47^ Thus, electrochemical analysis using Al_2_O_3_-NPs/MWCNTs/CPE revealed a dramatic increase in electrocatalytic activity.^48^ To the best of our knowledge, so far, no electrochemical method for the simultaneous electrocatalytic determination of TAM/SOL has been published.

The objective of the present work is to offer the first, simple, cheap, sensitive, rapid, and powerful electrochemical method to deal with the aforementioned serious challenges facing the simultaneous analysis of TAM/SOL in their challenging dosage form using Al_2_O_3_-NPs/MWCNTs/CPE. In addition to, providing an easy, rapid, and sensitive solution for the diagnosis of the fainting resulting from the TAM/SOL **first-dose phenomenon** by simultaneous analysis of TAM/SOL in biological fluids in nanomolar concentration with lower detection and quantification limit compared with the published methods in which the maximum concentration of TAM/SOL in plasma is out their linear range. In addition, it offers an alternative greener recycling procedure for the accumulated and environmentally polluting aluminum foil waste by synthesizing aluminum oxide nanoparticles from aluminum foil waste.

## 2. Experimental

### 2.1. Apparatus

All measurements of voltammetric techniques were done using Gamry Reference 3000™ instrument (potentiostat/galvanostat/ZRA model), the cell with three electrodes consisted of a working electrode (Al_2_O_3_-NPs/MWCNTs/CPE), a platinum wire from BAS (USA) as the auxiliary electrode, and the Ag/AgCl (3 M KCl) electrode as the reference electrode from BAS (USA). JENWAY 3510 digital pH meter. In addition, to analyze the synthesized samples, FT-IR spectrometer (IRaffinity-1, Shimadzu) was used to confirm the functional groups of the particles, the distribution of surface morphology and nanoparticle chemical composition were investigated *via* Helios Nanolab-400 scanning electron microscopy (SEM) linked to (EDX) the energy dispersive X-ray analysis and the determination of the nanoparticles' particle size was employed *via* (TEM) transmission electron microscopy (Hitachi H.7500). The synthesized samples were analyzed using X-ray diffraction (XRD; Rigaku Smart Lab), with Cu Kα radiation (*λ* = 1.54056 Å) for scattered X-ray at different angles from 20° to 70° with a step of 0.0195 at room temperature. Approximately 25 °C was used for all voltammetric measurements.

### 2.2. Chemicals and reagents

Standard TAM/SOL powders (99.86% and 99.95%, respectively, the purity was checked by applying the reported method^19^) and their commercial formulation (Tamsulin Plus®), B. no. R01230382, were supplied by Marcyrl Pharmaceutical Industries company (El Obour City, West Extension, Block 20005, Cairo, Egypt). Tamsulin Plus®, each tablet claimed to have 0.4 mg and 6 mg for TAM/SOL respectively.

All experimental solutions were prepared using ingredients of high analytical grade (Sigma-Aldrich, Germany). Britton Robinson (B–R) buffer has been obtained by adding an accurate equal concentration of (0.04 M boric acid : phosphoric acid : acetic acid) then titration with 0.2 M NaOH to get the required pH.^49^

### 2.3. Standard solutions

The Standard drug solutions of TAM/SOL each of 1.00 × 10^−2^ M were obtained (4.085 μg ml^−1^ and 3.625 μg ml^−1^ of TAM/SOL, respectively) by dissolving the amount of standard TAM/SOL, respectively, in methanol. The freshly working TAM/SOL solutions were prepared by appropriate stock solution dilution employing methanol. The obtained solutions were stable enough and can be used in a dark refrigerator bottle for about one week (5 °C).

### 2.4. Synthesis of Al_2_O_3_ nanoparticles

Using water, samples of aluminum waste were cleaned to get rid of any surface accumulated debris and then, drying in an oven for (4 h) in order to remove oxide layers, the sample surface pretreatment was carried out by gradually filling the surface. Then discarded aluminum foil (approximately 40.0 g) was progressively added to a solution of 56.6 ml HCl acid (36%) and the same amount of water until effervescence stopped generating aluminum chloride. Followed by filtration to get rid of impurities then cooled. Aluminum oxide precipitation is accomplished by slowly adding 1 M sodium carbonate, ensuring that all aluminum chloride has been changed to aluminum oxide, and then allowing it to settle for 1 hour after adding distilled water. Wash off the sodium chloride by decantation. In order to obtain aluminum oxide, a solution was filtered then and dried for (3–4 h) in an oven (100 °C). Finally, to obtain Al_2_O_3_ nanoparticles, the acquired samples are calcinated at 500 °C for 5 hours.^47^

### 2.5. Working electrodes

The CPE fabrication was done by a glassy mortar and pestle by a homogeneous mixing of a graphite powder (0.4 g) with paraffin oil (0.2 ml). The obtained homogenous paste was put into the electrode body nozzle then a smooth, shiny electrode surface was achieved by smoothing on filter paper. Then by pressing a copper wire into the paste, the connection with the apparatus is achieved.

Preparation of modified electrode was carried out by mixing of graphite powder (0.4 g) with MWCNTs (0.08 g) or/and Al_2_O_3_ nanoparticles powder (0.08 g), and paraffin oil (0.3 ml) employing a glassy mortar and pestle to obtain a homogenous paste to prepare MWCNTs/CPE and Al_2_O_3_-NPs/MWCNTs/CPE, and as with the bare electrode, the next procedures were repeated.

### 2.6. General procedure

To prepare the final desired concentrations, different aliquots of TAM/SOL standard solutions, respectively, were added and totally mixed with B–R (pH 4), and then for 6 min, a stream of inert nitrogen gas was used to deoxygenate the test solutions and then in a positive direction, voltammograms of square wave were obtained at a scan rate (0.1 V s^−1^) with an applied potential range (0: +1.4 V), pulse amplitude (0.02 V), and frequency (20 Hz) using both of bare & modified-carbon paste electrodes. The regression equations for TAM/SOL, respectively, were computed using a calibration curve of the concentration (ng ml^−1^) against peak current (*I*_p_).

### 2.7. Application to dosage form

Twenty (Tamsulin Plus®) tablets were accurately weighed. Subsequently, in a dry and clean mortar, the contents were finely crushed. Thereafter, a suitable equivalent portion of the obtained powder was transferred, and sufficient methanol was used to dissolve it by shaking then sonication for 15 min. Then filtration in a volumetric flask (100 ml), the solution was filtered. Then, the final volume was adapted with the same solvent. To prepare the final desired concentrations, different aliquots covering the concentration ranges of TAM/SOL, respectively, were added and totally mixed with buffer (pH 4). Simultaneous quantification of TAM/SOL in dosage form was achieved by direct proceeding of the developed method. Using the associated regression equation, the concentration of TAM/SOL was obtained, and the recovery (% *R*) was estimated.

### 2.8. Spiked human urine

Prior to the actual experiments, a non-smoking, drug-free, and healthy volunteer provided a blank urine sample. The urine samples spiked with different concentrations of TAM/SOL, respectively, then centrifugation at 5000 rpm for 10 min to get rid of any unexpected endogenous chemicals. Adequate volume of the upper clear supernatant was diluted with an adequate volume of B–R buffer (pH 4). Simultaneous determination of TAM/SOL in the urine was done by direct proceeding of the developed method. Using the associated regression equation, the concentration of TAM/SOL was obtained, and the recovery (% *R*) was estimated.

### 2.9. Spiked human plasma

Prior to the actual experiments, fresh samples of human plasma from the hospital of October 6 University, 6th of October City, Giza, Egypt with approval number is PRC-Ph-2204016. Plasma samples were diluted five times using buffer (pH 4) to diminish the matrix effects. Diluted plasma samples were spiked with appropriate different concentrations of TAM/SOL, respectively. Simultaneous determination of TAM/SOL in the plasma was done by direct proceeding of the developed method. Using the associated regression equation, the concentration of TAM/SOL was obtained, and the percent recovery (% *R*) was estimated.

### 2.10. Ethical statement

The study involving human participants biological samples and it was performed in strict accordance with the institutional ethical standards of the Helsinki Declaration of 1964 and its later amendments. It was approved by the local research ethical committee of October 6 University, 6th of October City, Giza, Egypt. The approval number is PRC-Ph-2204016. The samples were collected from the hospital of October 6 University, 6th of October City, Giza, Egypt. Postal code 12585. Healthy volunteers were fully informed verbally about the objectives, nature and possible risks of this study and a written informed consent was obtained from all the volunteers involved in the study.

## 3. Results and discussion

### 3.1. Modified electrode characterization

**Fig. 1:**
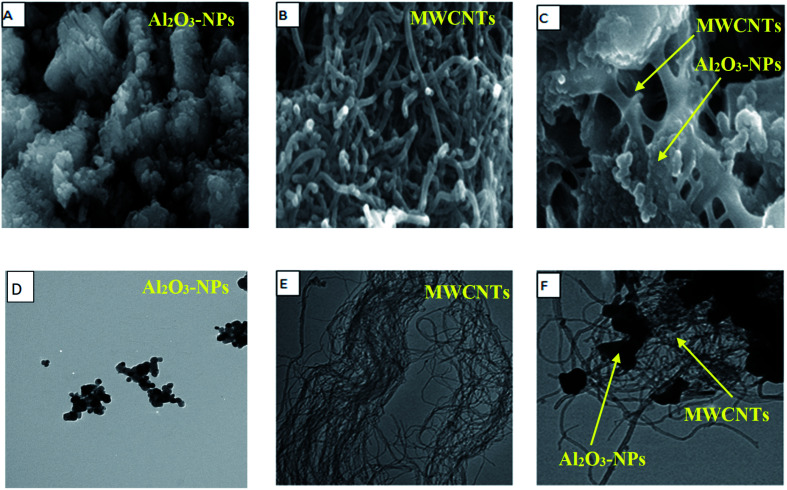
(A) SEM image, (B) MWCNTs SEM image, (C) Al_2_O_3_-NPs/MWCNTs SEM image, (D) Al_2_O_3_-NPs TEM image, (E) MWCNTs TEM image, (F) Al_2_O_3_-NPs/MWCNTs TEM image.

**Fig. 2:**
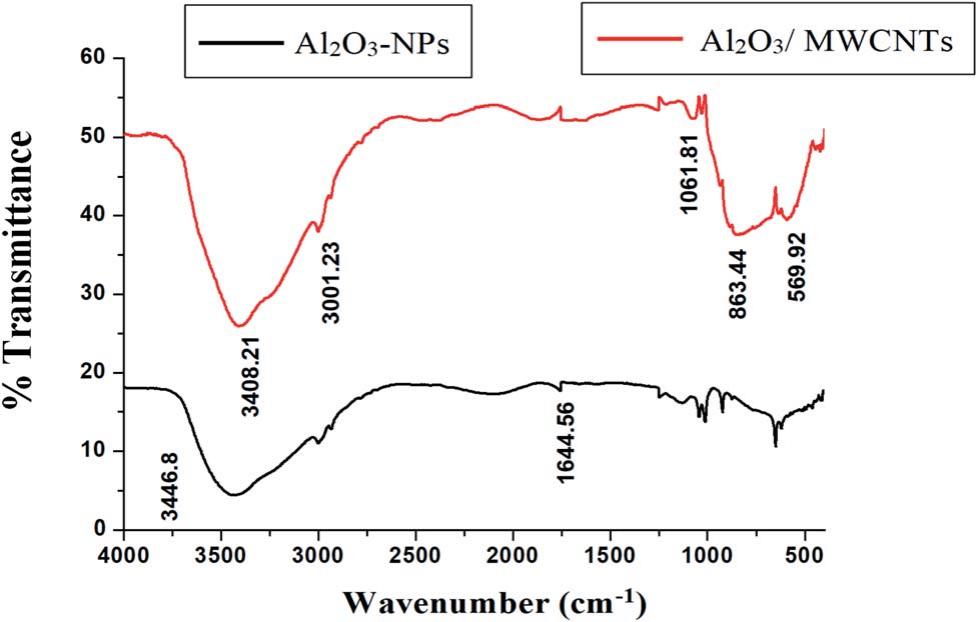
FTIR spectra of MWCNTs and Al_2_O_3_-NPs/MWCNTs.

**Fig. 3:**
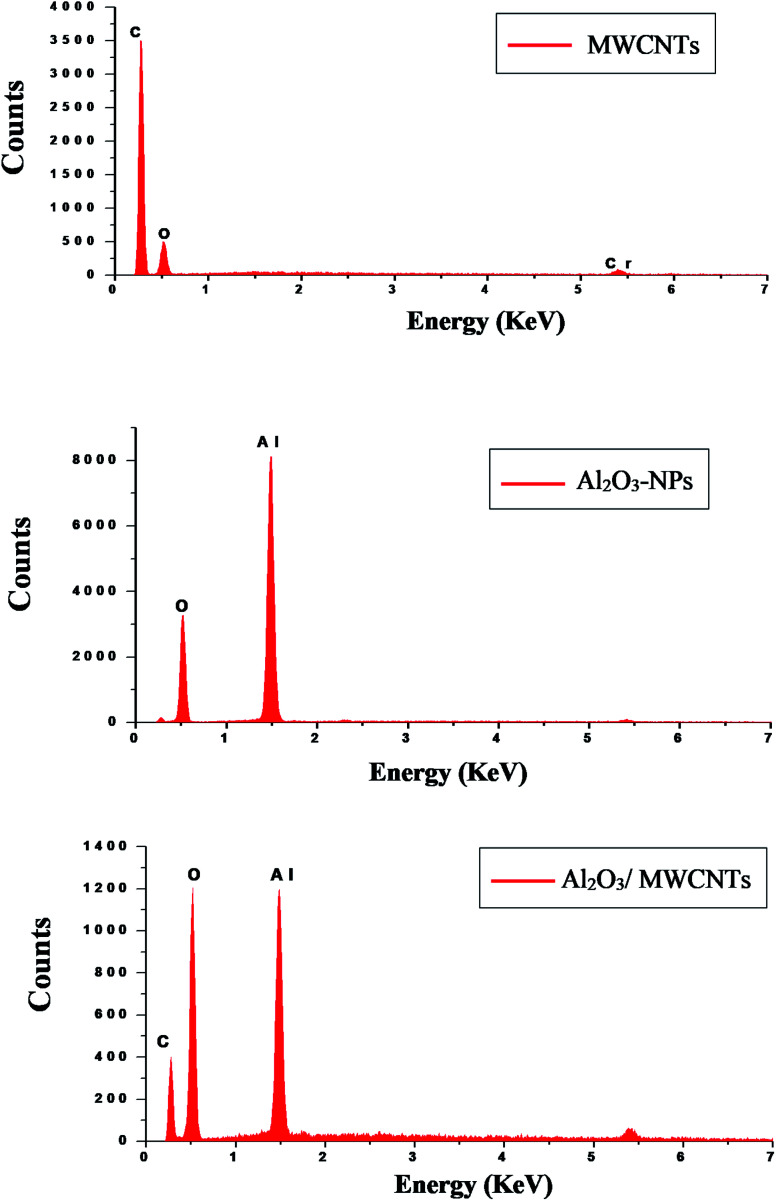
EDX patterns of MWCNTs, Al_2_O_3_-NPs, and Al_2_O_3_-NPs/MWCNTs.

**Fig. 4:**
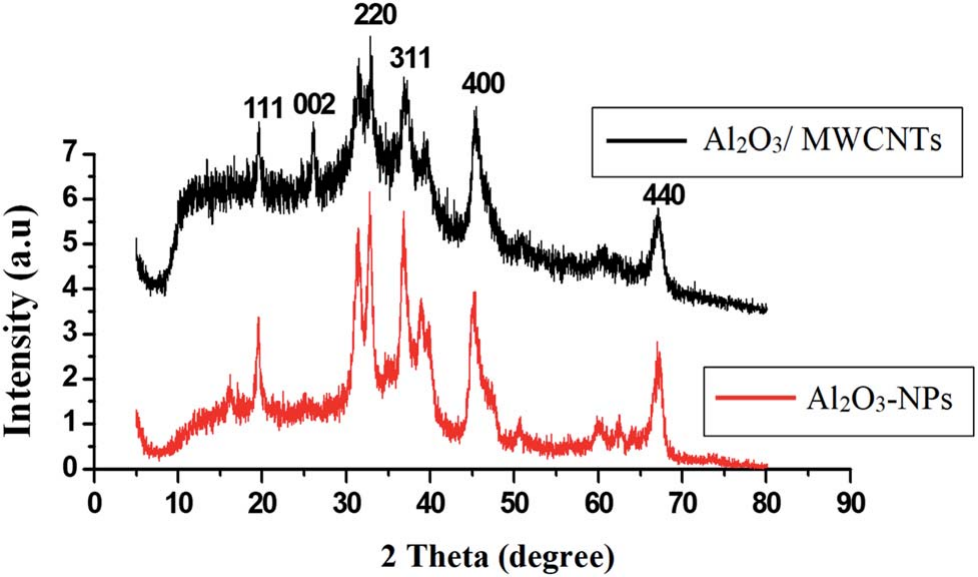
XRD patterns of Al_2_O_3_-NPs and Al_2_O_3_-NPs/MWCNTs.

SEM, EDX, TEM, XRD and FTIR techniques were employed for the modified electrode characterization. SEM, as shown in Fig. 1, was used to characterize the morphologies of the Al_2_O_3_-NPs/MWCNTs electrode. SEM image showed irregular rough spherical surfaces of Al_2_O_3_-NPs that appeared equiaxed with uniform distribution and good spherical shape results (roundness aggregate above 80%) as shown in Fig. 1A which are characteristic for Al_2_O_3_ nanoparticles.^50^ On the other hand, numerous groves of the MWCNTs as shown in Fig. 1B, offer an exceptional electro-active surface area compared with bare CPE.^51^ Interestingly, Al_2_O_3_ nanoparticles' dispersion on the MWCNTs surface is evident from the TEM image as shown in Fig. 1F, where particle size is about (5–10 nm). Furthermore, the infrared spectrum contributes to obviously understanding the modified electrode surface chemistry as the FTIR bands considered as fingerprints for molecules as shown in Fig. 2 where the characteristic peaks at (500–1000 cm^−1^) correspond to (O–Al–O) bonds which are characteristic for Al_2_O_3_ nanoparticles.^52^ On the other hand, the clear sharp peak around (1644.56 cm^−1^) is distinguishing for (aromatic rings C=C) bonds of the skeleton of carbons. The –OH groups broad peak at (3366–3420 cm^−1^) refer to (–OH) functional groups of MWCNTs surface.^51^Fig. 3 demonstrates the EDX spectra of MWCNTs, Al_2_O_3_ NPs, and modified Al_2_O_3_-NPs/MWCNTs. The EDX spectrum of MWCNTs demonstrates carbon and oxygen as the only basic elementary components of MWCNTs, on the other hand, the EDX spectrum of Al_2_O_3_NPs demonstrates the presence of aluminum and oxygen. Thus, the presence of aluminum, carbon, and oxygen in modified Al_2_O_3_-NPs/MWCNTs nanocomposites EDX spectrum with a remarkable decrease in peak intensity of carbon and aluminum compared to EDX spectrum of pure MWCNTs and Al_2_O_3_-NPs, respectively, is the proof for surface modification.^53^ The XRD patterns of Al_2_O_3_-NPs, peaks are compatible with the standard JCPDS data (card no. 79-1558) and confirm the formation of Al_2_O_3_ NPs. The distinct diffraction peaks located at different diffraction angles 2*θ* were 19.64°, 31.62°, 36.75°, 45.32°, and 67.33° have been keenly indexed for pure Al_2_O_3_-NPs,^54^ as shown in Fig. 4. Upon incorporation of MCNTs with Al_2_O_3_-NPs, the most diffraction peaks for Al_2_O_3_-NPs were broaden and the peak at 26.2 corresponding to (002) plane in the XRD pattern of Al_2_O_3_-NPs/MWCNTs indicates the presence of MWCNTs along with the Al_2_O_3_-NPs. Furthermore, the intensity of this peak is very low when compared to Al_2_O_3_-NPs peaks which could be attributed to the low diffraction intensity of MWCNTs when compared to Al_2_O_3_-NPs.

### 3.2. The electrodes electro-active surface area

High surface area is a very important feature of the electrodes as it leads to an increase in electrochemical performance by augmentation of charge carriers. The electro-active area for each electrode could be calculated using the Randles–Ševčík equation^55^ with the aid of different scan rates cyclic voltammetric determination of K_4_Fe(CN)_6_ (1.0 × 10^−3^ M) in KCl (0.1 M). The Randles–Ševčík equation was employed as the following:*I*_pa_ = 2.69 ×10^5^*n*^3/2^*AD*^1/2^*Cν*^1/2^,where (*I*_pa_) is the anodic peak maximum current in amps, (*n*) is the number of transferred electrons in the redox reaction, (*A*) is the surface area of the electrode (cm^2^), (*D*) is the diffusion coefficient (cm^2^ s^−1^), (*C*) is the concentration (mol cm^−3^) of K_4_Fe(CN)_6_, and (*ν*) refers to scan rate (V s^−1^). (*n*) equal 1 and (*D*) equal 7.63 × 10^−6^ cm^2^ s^−1^.

**Fig. 5:**
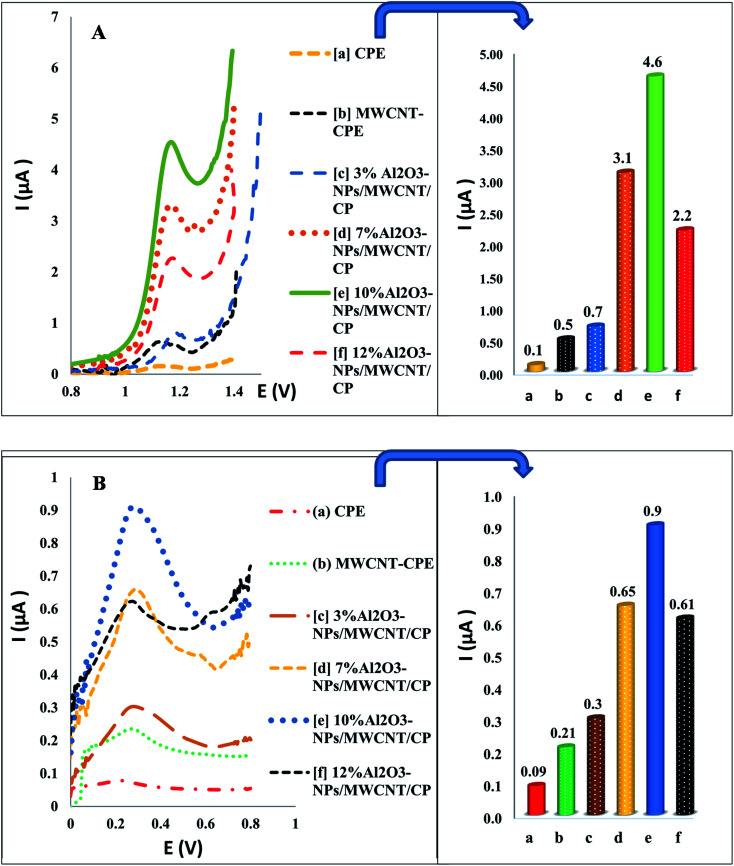
The Al_2_O_3_-NPs contents and different electrodes effect on oxidation peak height of TAM (A) and SOL (B) using SWV.

The effective electro-active surface areas of different modified electrodes were found as 0.25 cm^2^ for Al_2_O_3_-NPs (10%, w/w)/MWCNTs/CP, 0.094 cm^2^ for MWCNTs/CP and 0.03 cm^2^ for CPE. Interestingly, compared to other examined electrodes, Al_2_O_3_ (10%, w/w)/MWCNTs/CPE possess the most effective electro-active surface area. The effects of aluminum oxide nanoparticles content were studied, and the maximum oxidation peak height of TAM/SOL was detected by 10% of aluminum oxide nanoparticles as shown in Fig. 5.

### 3.3. The cyclic voltammetric behavior of TAM/SOL

**Fig. 6:**
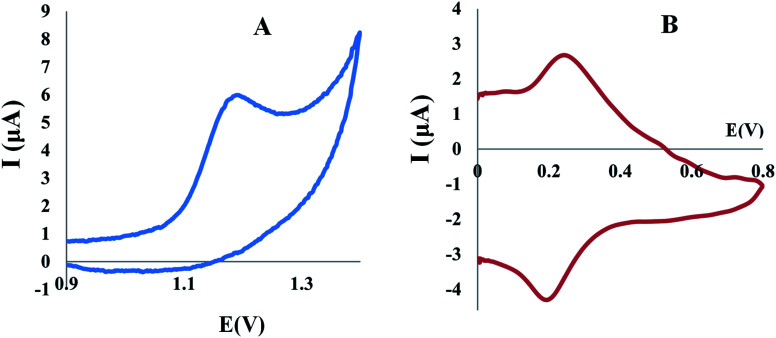
(A) Cyclic voltammogram of 1.00 × 10^−3^ M TAM, (B) cyclic voltammogram of 1.00 × 10^−3^ M SOL at Al_2_O_3_-NPs/MWCNTs/CPE in BR buffer (0.04 M) pH 4.0 and scan rate of 100 mV s^−1^.

The cyclic voltammogram of TAM/SOL in B–R buffer (pH 4) assisted by modified electrodes as shown in Fig. 6 showed one irreversible oxidation anodic peak for TAM and it also observed that, in the reverse scan, there is no reduction peak, while for SOL, there are two peaks, oxidation peak, and reduction peak. For TAM/SOL, the anodic peak current assisted by Al_2_O_3_-NPs/MWCNTs/CPE has considerably enhanced and was extensively higher than the current based on the CPE and MWCNTs/CP electrodes. On the Al_2_O_3_-NPs/MWCNTs/CPE, the oxidation anodic peak appeared at about 1.2 and 0.24 V for TAM and SOL respectively, with considerable improvement in the peak current due to the larger active area and electrocatalytic activity.

### 3.4. Optimization of the method

Depending on buffer changing, several pHs, and several scan rates, the experimental conditions were optimized. Various buffers, including (Britton–Robinson, phosphate, borate, acetate, and citrate), have been examined. The Britton–Robinson buffer had extensively characteristic sharp peaks. TAM/SOL electro-oxidation behavior was studied with different pH and scan rates. Our findings indicate that the optimum peak was acquired at pH 4 and 100 mV s^−1^.

#### 3.4.1. Effect of pH on electrodes response

**Fig. 7:**
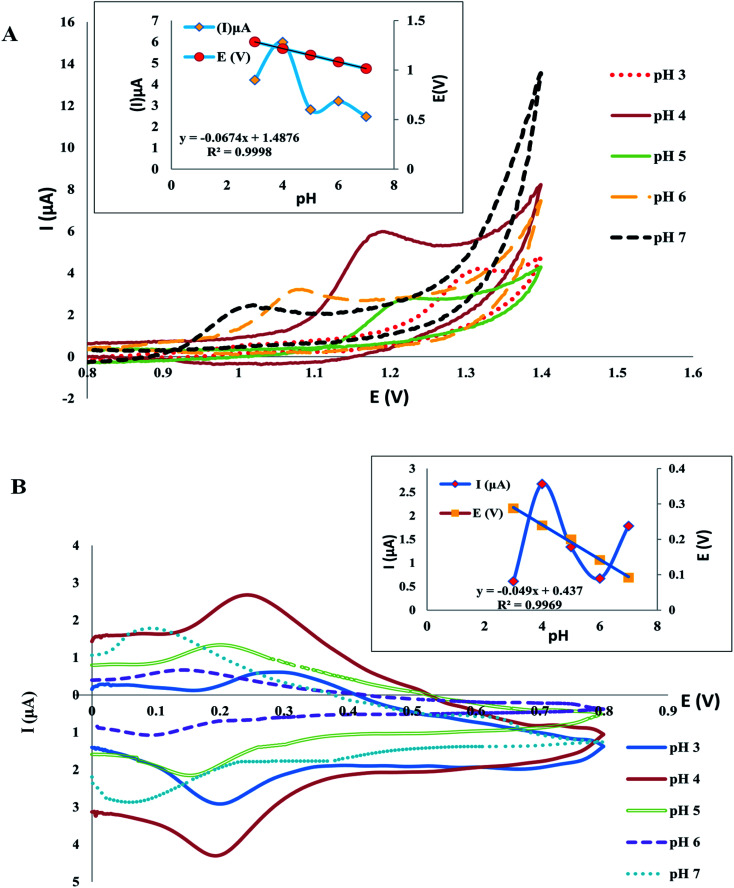
The pH effect on the potential and peak current of TAM (A) and SOL (B) in BR buffer (0.04 M) pH 4.0.

The response of the electrodes extensively depends on the buffering solution and medium pH as the oxidation potential and peak current are based on the pH value of the solution thus, medium pH has a crucial role in the electrochemical behavior of electrodes. The CV was used for the investigation of pH influence on the present response, using Britton Robinson buffers (0.04 M) with pH (3–7). The results of plotting pH against peak current (*I*_p_) indicated that the optimum high peak current was acquired with pH 4 for both drugs Fig. 7, in addition, the results of plotting pH against peak potential *E* (V) indicated that *E* was negatively affected by an increase in pH values, confirming the participation of protons in the reaction of oxidation of both drugs Fig. 7, and the following equations were used to estimate the linear relationship of TAM and SOL between *E* and pH:*E* (V) = −0.0674pH + 1.487, for TAM*E* (V) = −0.049pH + 0.437, for SOLthe slope of the linear relationship between *E* and pH of both drugs was found to be approximately equal to the Nernst equation, as the slope of TAM and SOL was −0.657 and −0.049 mV pH^−1^ respectively, showing that, the number of protons and electrons involved in the reaction of the electrode is the same.^51^

#### 3.4.2. Effect of scan rate

**Fig. 8:**
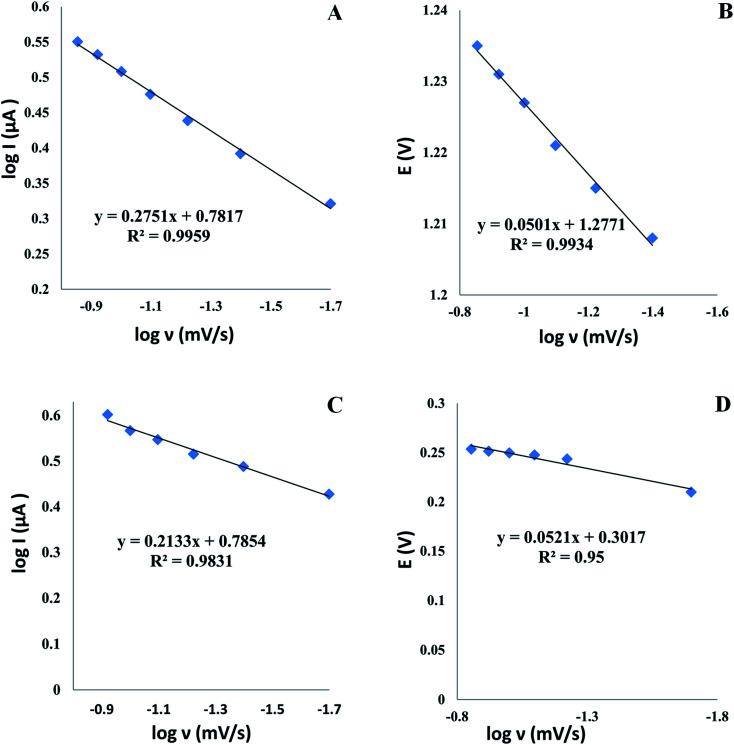
The log scan rate (*ν*) *versus* the log peak current *I*_p_ and the potential E of TAM (A and B) and SOL (C and D) at Al_2_O_3_-NPs/MWCNTs/CPE.

To acquire greater insight into the TAM/SOL redox reactions at the modified electrode, the study of the scan rates effect on the current of oxidation peaks was done for each drug in a pH 4.0 depending on cyclic voltammetry, which interestingly helped in demonstrating the kinetic mechanism and many investigations of the redox system, for example, scan rates studies were carried out to establish whether an adsorption or diffusion process was used for controlling the electrochemical reaction. For both TAM and SOL, it was clear that we deal with the diffusion-controlled electro-oxidation process as by raising the scan rate, the peak current increased in the range of 0.02 to 0.12 V s^−1^ in addition to the linear relationship correlate between logarithm current (log *I*_p_) *versus* logarithm scan rate (log *ν*) Fig. 8, and the slope values of both drugs, 0.2751 and 0.2354 for TAM and SOL respectively, which is approximately near to diffusion-controlled theoretical value (0.5) as shown in the following equations:log *I*_p_ = 0.2751 log *ν* + 0.7817, for TAMlog *I*_p_ = 0.2133 log *ν* + 0.7854, for SOLfor further study of the electron transfer kinetics, we plotted potential *E*_p_ (V) *versus* logarithm scan rate (log *ν*) Fig. 8 to obtain the following equations:*E*_p_ (V) = 0.0501 log *ν* + 1.2771, for TAM*E*_p_ (V) = 0.0521 log *ν* + 0.3017, for SOLthen the reaction electrochemical electrons number (*n*) was determined depending on (Laviron equation^56^) and the slopes of the aforementioned equations.

**Scheme 1:**
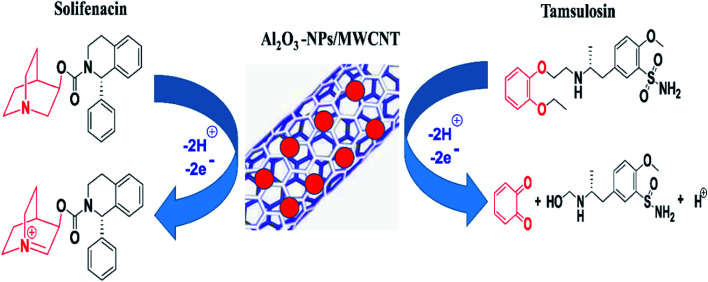
Proposed oxidation mechanisms of tamsulosin & solifenacin and sensor configuration.

Laviron equation:
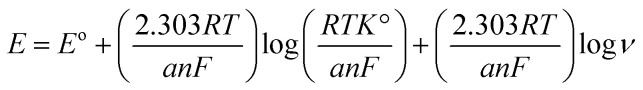
from the aforementioned equations, the slopes equal 0.0501 and 0.0521 for TAM and SOL respectively, thus (*αn*) was calculated to be 1.158 and 1.113 for TAM and SOL respectively. Due to the TAM/SOL irreversible electro-oxidation, (*α*) was equal to 0.5 and (*n*) was calculated to be 2.31 (≈2) and 2.22 (≈2) for TAM and SOL respectively that is agreed with the suggested electro-oxidation mechanisms of TAM/SOL as shown in Scheme 1.

#### 3.4.3. Chronoamperometric studies

Chronoamperometry as shown in (Fig. S1 and S2†) is used to estimate the diffusion coefficient of TAM and SOL. Ideally, the chronoamperograms were derived using four different concentrations of each drug using Al_2_O_3_-NPs/MWCNTs/CPE over five seconds at a constant potential of 1.2 and 0.24 V for TAM and SOL respectively in pH 4 using BR buffer, then *via* plotting the relationship between current (*I*) *versus* (*t*^−1/2^), it was observed that there was a linear relationship between the two variables Fig. S1.† Consequently, the calculation of the diffusion coefficient (*D*) was done using the Cottrell equation depending on the slopes obtained by plotting the TAM and SOL concentrations against the resulting slopes of the aforementioned relationship^57^ Fig. S2.† The diffusion coefficients for TAM and SOL were found to be 33.46 × 10^−5^ and 22.44 × 10^−5^ cm^2^ s^−1^, respectively.

### 3.5. Determination of TAM and SOL in pure forms

**Fig. 9:**
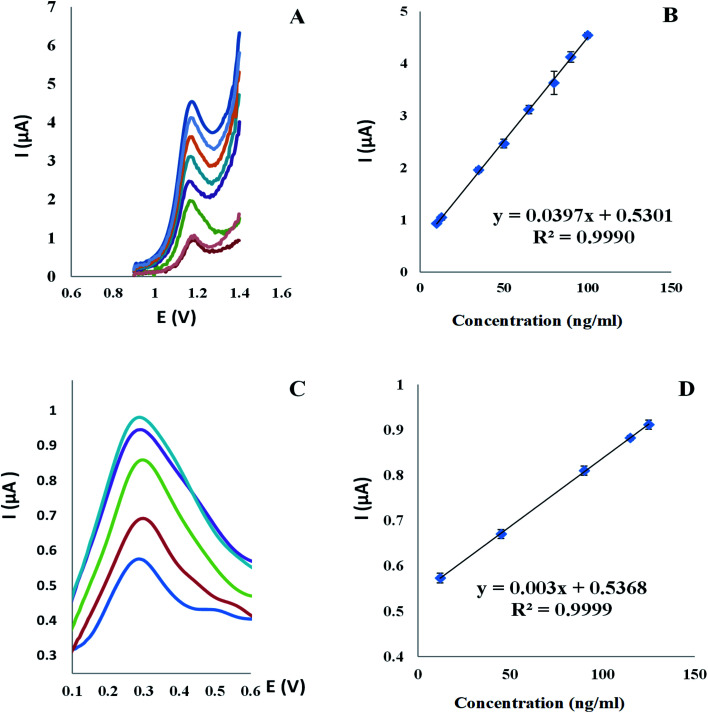
(A) Square wave voltammograms and (B) the corresponding calibration curve for TAM concentrations range (10 to 100 ng ml^−1^) and (C) square wave voltammograms and (D) the corresponding calibration curve for SOL concentrations range (12 to 125 ng ml^−1^) at Al_2_O_3_-NPs/MWCNTs/CPE in BR buffer (0.04 M) pH 4.0 and scan rate of 100 mV s^−1^.

Voltammograms of square wave on the Al_2_O_3_-NPS/MWCNTs/CPE were obtained at a scan rate (0.1 V s^−1^) with an applied potential range (0.0: +1.4 V). Interestingly, the oxidation peak of TAM appeared at 1.18 V with high current intensity, measured by microampere [μA], while the oxidation peak of SOL appeared at 0.29 V with approximately low current intensity, measured by nanoampere [nA], thus this wide variation in current intensity between TAM and SOL contributes in easy, accurate, sensitive and extremely selective TAM/SOL simultaneous quantification, as shown in Fig. 9. Concentration and peak current have a linear relationship with the concentration of 10–100 ng ml^−1^ for TAM and 12–125 ng ml^−1^ for SOL. Table 1 shows statistical parameters.

**Table 1:** Validation data of TAM/SOL at the Al_2_O_3_-NPs/MWCNTs/CPE

Parameters	TAM	SOL
Linearity (ng ml^−1^)	10–100	12–125
Intercept	0.530	0.537
Slope	0.039	0.003
Correlation coefficient (*r*)	0.999	0.999
Accuracy	99.28%	100.03%
Precision (% RSD)		
Repeatability^a^ (% RSD)	0.69	0.88
Intermediate precision^b^ (% RSD)	1.25	1.15
Robustess^c^ (mean ± % RSD)	99.55 ± 1.63	100.48 ± 0.45
LOD (ng ml^−1^)	2.69	3.25
LOQ (ng ml^−1^)	8.15	9.85

a Average of TAM/SOL three different concentrations were repeated three times within the day.

b Average of TAM/SOL three different concentrations were repeated three times in three days.

c Variation in method parameters such as pH of the sample and measurement resting period.

### 3.6. Method validation

The International Conference on Harmonization (ICH) guidelines for method validation was followed.^58^

#### 3.6.1. Linearity and range

TAM was found to be linear between 10–100 ng ml^−1^ and SOL was found to be linear between 12–125 ng ml^−1^ as shown in Fig. 9.

#### 3.6.2. Limit of detection and quantification

The limit of detection (LOD) and limit of quantitation (LOQ) describe the minimum concentration that an analytical method can accurately quantify which is the proof of suggested method sensitivity as shown in Table 1. In comparison to the reported methods, the suggested method was successful in estimating TAM/SOL with a lower limit of detection and high sensitivity, as shown in Table 2.

**Table 2:** Comparison of the suggested voltammetric method and the reported methods

Analytical method	TAM		SOL		Application	Reference
	LOD	LOQ	LOD	LOQ		
RP-HPLC method (μg ml^−1^)	0.05	0.1	0.04	0.14	Tablets	19
HPLC method (μg ml^−1^)	0.014	0.043	0.075	0.221	Tablets	20
HPTLC method (μg ml^−1^)	0.03	0.10	0.30	1.00	Capsules	18
Derivative synchronous emission spectroscopy (μg ml^−1^)	0.210	0.639	2.64	8.0	Tablets	15
Spectrofluorimetric determination (μg ml^−1^)	0.13	0.40	0.60	1.81	Tablets and capsules	14
First order derivative spectrophotometric(μg mL^−1^)	0.101	0.308	1.054	3.196	Tablets	16 and 17
ACM (μg ml^−1^)	1.25	3.80	19.22	58.25	Tablets	16 and 17
D1 (μg ml^−1^)	1.34	4.05	24.60	74.55	Tablets	16 and 17
Square wave voltammetry (Al_2_O_3_-NPs/MWCNTs/CPE) (ng ml^−1^)	2.69	8.15	3.25	9.85	Tablets, spiked plasma, and urine	Our present study

#### 3.6.3. Accuracy

Accuracy as mean percent recovery (% *R*) was calculated. The satisfactory % *R* for TAM/SOL confirmed the good accuracy of the proposed SWV method as shown in Table 1.

#### 3.6.4. Precision

Repeatability (intra-day); in a single day, three replicate determinations of three chosen concentrations of TAM/SOL were conducted. The % relative standard deviation (% RSD) was used to express the suggested method precision, where the (% RSD) satisfactory values imply the accepted repeatability of the suggested method Table 1.

Intermediate precision: for three days, the same concentrations was measured (inter-daily) employing the same procedures, and % RSD was less than 2%, proofing perfect intermediate precision of the suggested method Table 1.

#### 3.6.5. Robustness

The extensive ability of the analytical technique to remain unaffected by minor variations in the conditions of the method such as pH (4.0 ± 0.1) as well as each measurement resting period (5 s ± 1 s) is known as robustness.^59^ No remarkable variations were noted in the results, reflecting the robustness and method's reliability Table 1.

#### 3.6.6. Reproducibility, reusability, and stability

The electrode reproducibility was investigated as shown in Fig. S3† by fabricating five Al_2_O_3_-NPs/MWCNTs/CP electrodes under identical conditions. With a % RSD of 1.8, all the electrodes gave nearly identical responses, indicating optimum electrodes reproducibility. The reusability of the electrode was tested by comparing the voltammetric responses of the used electrode and the freshly prepared electrode (more than ten times). Both electrodes responded in the same way, indicating that the electrode can be reused. Furthermore, within 30 days, the fabricated electrode stability was tested by measuring the resulting current produced using the proposed method, showing a high level of reliability as shown in Fig. S4.†

### 3.7. Application to pharmaceutical dosage form

TAM and SOL were successfully quantified in a commercial dosage form as shown in Table 3. Furthermore, to determine the validity of the suggested SWV method, the standard addition technique was carried out, as shown in Table 3. Moreover, using *F* value and Student's *t*-test for statistical comparison between the suggested SWV method and the reported one signalized that, there are no significant differences as shown in Table 4.

**Table 3:** Determination of TAM/SOL at the Al_2_O_3_-NPs/MWCNTs/CPE in their dosage form and standard addition technique application

Tablets	Drug	% found ± SD^a^	Standard addition technique		% recovery^a^
			Added (ng ml^−1^)	Founded (ng ml^−1^)	
Tamsulin Plus® claimed to have 0.4 mg and 6.0 mg for TAM/SOL respectively	TAM	100.06 ± 0.286	15	14.9	99.33
			30	29.8	99.39
			40	40.1	100.19
			50	49.6	99.15
			Mean ± % RSD		99.52 ± 0.465
	SOL	100.05 ± 0.390	45	44.26	98.36
			55	54.69	99.45
			70	70.49	100.70
			80	80.93	101.17
			Mean ± % RSD		99.92 ± 1.26

a *n* = 3.

**Table 4:** Statistical comparison between the suggested and reported HPLC method

Parameters	Suggested method		Reported method^19^	
	TAM	SOL	TAM	SOL
Mean^a^	100.13	100.21	100.10	100.81
SD	0.127	0.281	0.22	0.52
Variance	0.016	0.079	0.048	0.270
*N*	5	5	5	5
Student's *t*-test (2.306)^b^	0.229	1.821	—	—
*F*-test (6.39)^a^	3.001	3.424	—	—

a *n* = 5.

b At *P* = 0.05.

### 3.8. Application to spiked human plasma and urine

TAM and SOL were successfully quantified in biological fluids. Once orally taken, TAM and SOL are approximately completely absorbed (above 90%) to reach peak plasma concentration of TAM equal (10.1 ng ml^−1^) following breakfast and (17.1 ng ml^−1^) during the fasting while for SOL equal (24.0 and 40.6 ng ml^−1^, respectively), with approximately 10% of the dosage is excreted unaltered in the urine,^12,13^ so that, the reported methods fail to quantify the maximum concentration of TAM/SOL in plasma and urine as the *C*_max_ is out their linear range, which explains the need for the proposed method. Interestingly, the proposed method showed perfect percentage recoveries of TAM and SOL in spiked plasma and urine as shown in Table 5 giving proof for the validity of the suggested method which can be employed without any interference for the estimation of TAM and SOL in spiked plasma and urine samples.

**Table 5:** Accuracy of TAM/SOL quantification at Al_2_o_3_-NPs/MWCNTs/CPE in spiked plasma and urine samples

Biological fluid	Drug	Added conc. (ng ml^−1^)	Found conc. (ng ml^−1^)	% recovery^a^	% RSD
Plasma	TAM	65	66.63	102.51	2.00
		90	93.07	103.41	1.99
		100	101.17	101.17	0.90
	SOL	45	43.33	96.29	1.87
		115	112.77	98.06	1.40
		125	129.50	103.60	0.81
Urine	TAM	50	50.90	101.81	1.53
		80	82.30	102.88	1.40
		100	103.11	103.11	1.98
	SOL	12	11.73	97.78	1.77
		90	92	102.22	0.69
		100	99.08	99.08	1.77

a *n* = 3.

## 4. Conclusions

In this work, the developed validated square wave voltammetric technique has been successfully presented as a first, powerful, simple, selective, sensitive, and precise method for simultaneous quantitative estimation of TAM and SOL in dosage forms having diverged concentration ranges and biological fluids having very low peak plasma concentration (*C*_max_) with satisfactory results using Al_2_O_3_-NPs/MWCNTs/CPE. The use of modified electrode increased the process of electron transfer as well as improved the electrode active surface area therefore, very sensitive results with low detection limits were acquired. The suggested methods can be confidently ranked between accurate and sensitive methods in respect of the results and validation parameters recorded in this paper. The high accuracy, precision, specificity, and sensitivity of the developed methods offer the chance to be employed in routine quality control analysis of TAM and SOL in laboratories that do not possess chromatographic instruments. Furthermore, the reported results in this paper offer an outlook for exploiting the voltammetric techniques for the quantification of pharmaceutical products having diverged concentration ranges and very low *C*_max_ by using simple, and inexpensive instruments regarding the number of samples, the number of interfering components.

## Conflicts of interest

There are no conflicts of interest to declare.

## Supplementary Material

RA-012-D2RA01962K-s001
