# Supplementary material for: Innovative electrochemical electrode modified with Al2O3 nanoparticle decorated MWCNTs for ultra-trace determination of tamsulosin and solifenacin in human plasma and urine samples and their pharmaceutical dosage form
Source: RSC Adv. 2022 Jun 13;12(27):17536–49. doi: 10.1039/d2ra01962k (PMC9192163; doi:10.1039/d2ra01962k)

Electronic Supplementary Material (ESI) for RSC Advances.
This journal is © The Royal Society of Chemistry 2022

Figure S1: (A) Chronoamperograms of TAM at Al_2O_3-NPs/MWCNTs/CPE in BR buffer (0.04 M) pH 4.0, containing: 10, 50, 70 and 100 ng/ml TAM. (B) The plot of I (μA) against $t^{-1/2}$ **($S^{-1/2}$)** for different concentrations of TAM. (C) The plot of the resulted slopes in (B) against the concentrations of TAM.

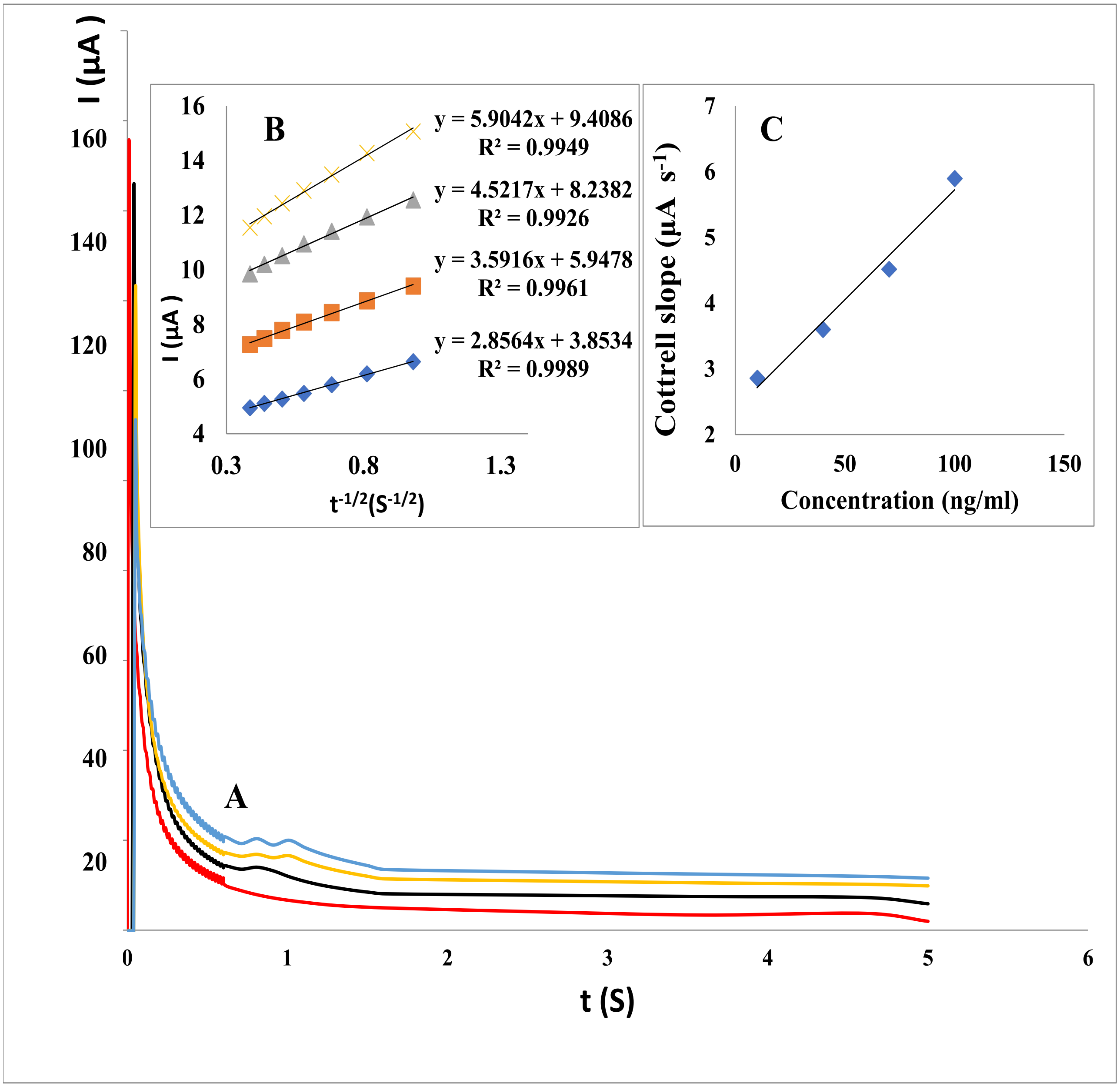

Figure S2: (A) Chronoamperograms of SOL at Al_2O_3-NPs/MWCNTs/CPE in BR buffer (0.04 M) pH 4.0, containing: 10, 70, 50 and 125 ng/ml SOL. (B) The plot of I (μA) against $t^{-1/2}$ ($S^{-1/2}$) for different concentrations of SOL. (C) The plot of the resulted slopes in (B) against the concentrations of SOL.

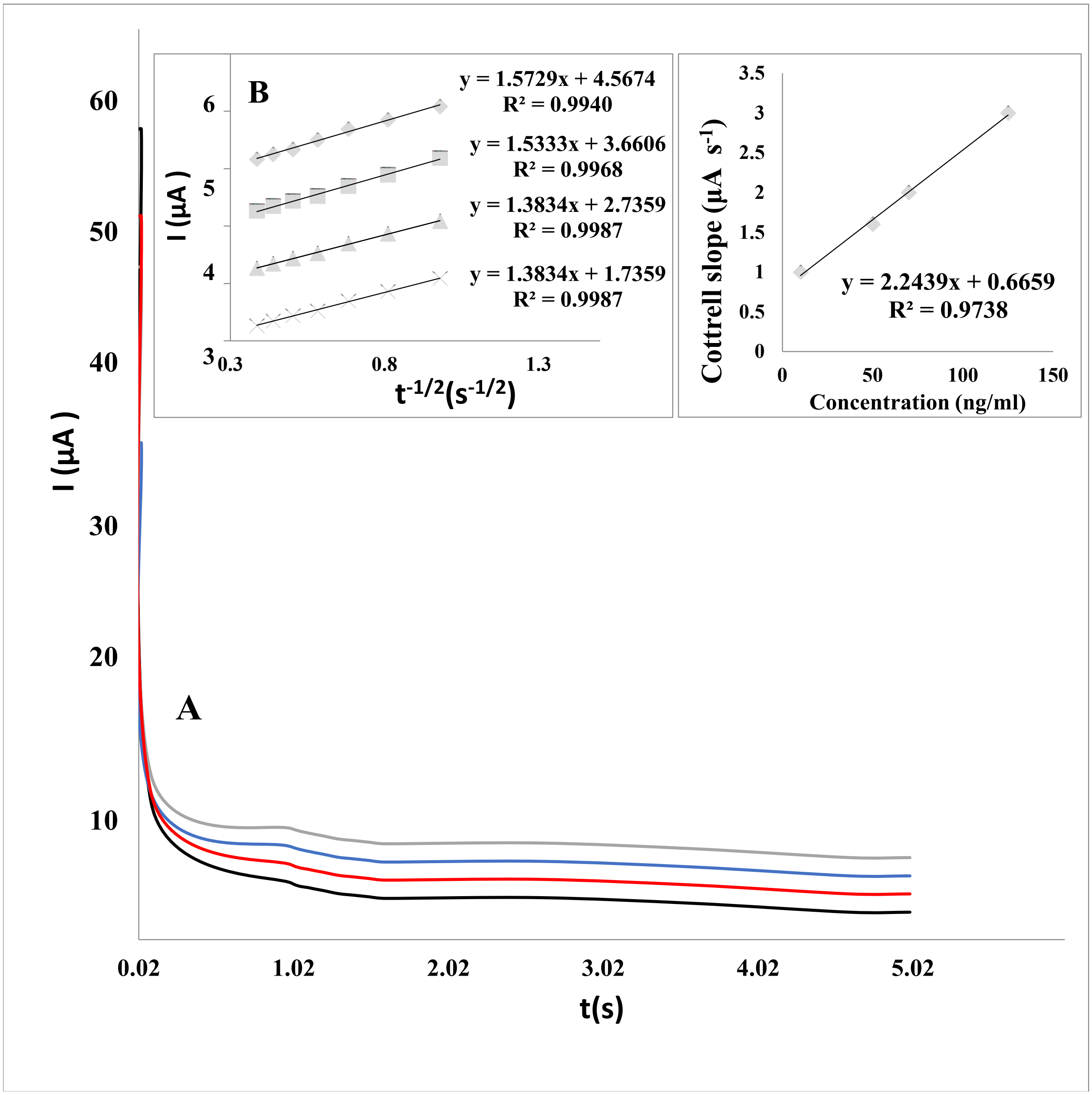

Figure S3: Reproducibility of Al_2O_3-NPs/MWCNTs/CPE in BR buffer pH 4.0, containing 100 ng/ml TAM (A) and 125 ng/ml SOL(B)

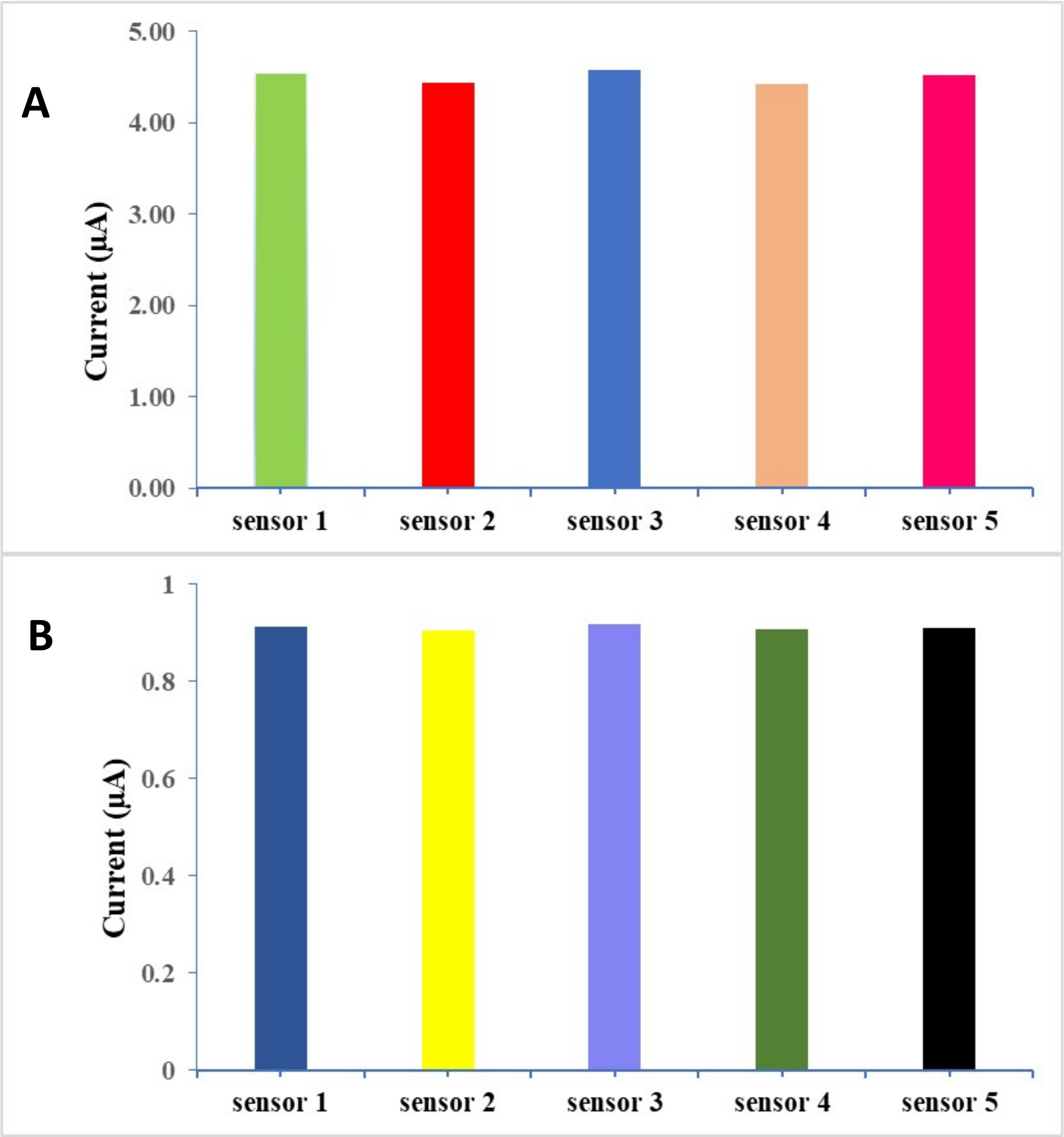

Figure S4: Stability of Al_2O_3-NPs/MWCNTs/CPE in BR buffer pH 4.0, containing 100 ng/ml TAM (A) and 125 ng/ml SOL(B)

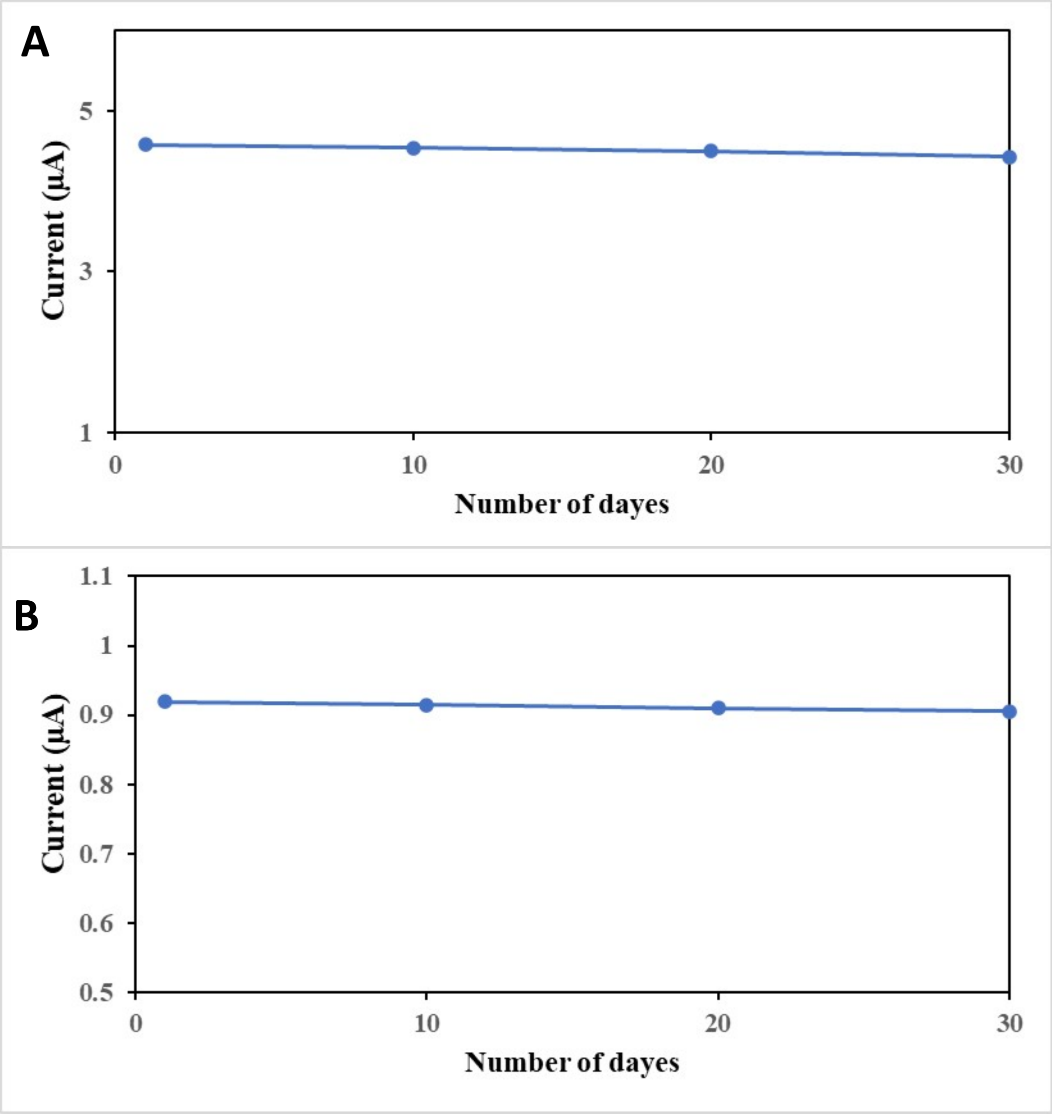

Figure S5: Square wave voltammogram for TAM and SOL at Al_2O_3-NPs/MWCNTs/CPE in BR buffer (0.04 M) pH 4.0 and scan rate of 100 mV/s.

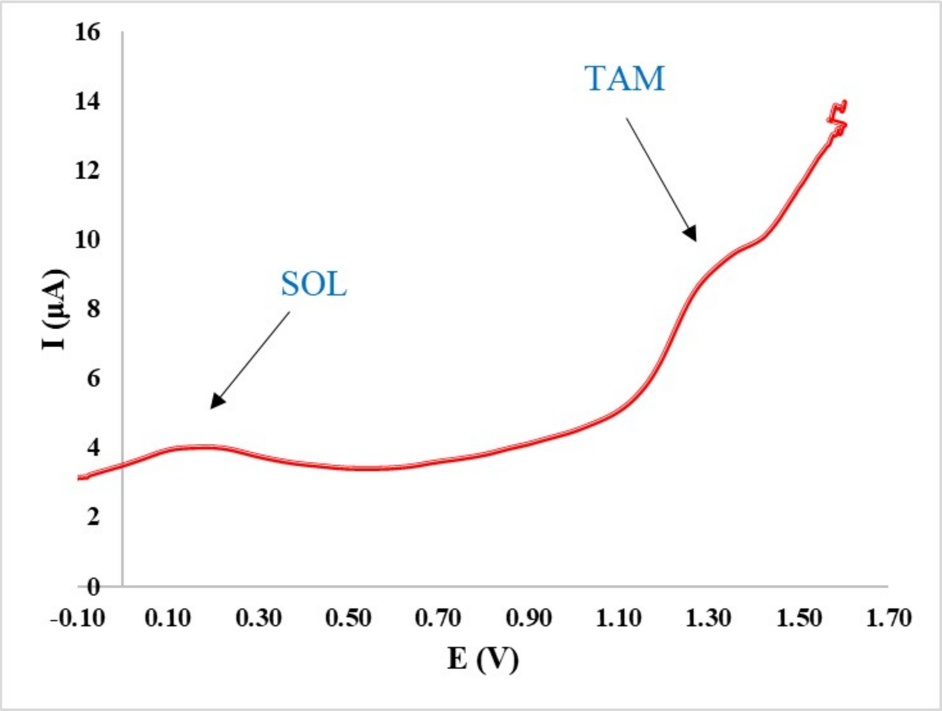

Supplement: RA-012-D2RA01962K-s001 [file RA-012-D2RA01962K-s001.pdf]
